# hGC33-Modified and Sorafenib-Loaded Nanoparticles have a Synergistic Anti-Hepatoma Effect by Inhibiting Wnt Signaling Pathway

**DOI:** 10.1186/s11671-020-03451-5

**Published:** 2020-11-26

**Authors:** Jing Shen, Wenpeng Cai, Yongfang Ma, Ruyue Xu, Zhen Huo, Li Song, Xinyin Qiu, Yinci Zhang, Amin Li, Weiya Cao, Shuping Zhou, Xiaolong Tang

**Affiliations:** 1 Medical School, Anhui University of Science and Technology, Huainan, 232001 China; 2 Wuhu Research Institute, Anhui University of Science and Technology, Huainan, 232001 China

**Keywords:** Glypican-3, Hepatocellular carcinoma, Targeted therapy, Wnt signal

## Abstract

Delivery of tumor-specific inhibitors is a challenge in cancer treatment. Antibody-modified nanoparticles can deliver their loaded drugs to tumor cells that overexpress specific tumor-associated antigens. Here, we constructed sorafenib-loaded polyethylene glycol-b-PLGA polymer nanoparticles modified with antibody hGC33 to glypican-3 (GPC3 +), a membrane protein overexpressed in hepatocellular carcinoma. We found that hGC33-modified NPs (hGC33-SFB-NP) targeted GPC3^+^ hepatocellular carcinoma (HCC) cells by specifically binding to GPC3 on the surface of HCC cells, inhibited Wnt-induced signal transduction, and inhibited HCC cells in G0/1 by down-regulating cyclin D1 expression, thus attenuating HCC cell migration by inhibiting epithelial–mesenchymal transition. hGC33-SFB-NP inhibited the migration, cycle progression, and proliferation of HCC cells by inhibiting the Ras/Raf/MAPK pathway and the Wnt pathway in tandem with GPC3 molecules, respectively. hGC33-SFB-NP inhibited the growth of liver cancer in vivo and improved the survival rate of tumor-bearing mice. We conclude that hGC33 increases the targeting of SFB-NP to HCC cells. hGC33-SFB-NP synergistically inhibits the progression of HCC by blocking the Wnt pathway and the Ras/Raf/MAPK pathway.

## Introduction

Glypican3 (GPC3) is a heparan sulfate proteoglycan expressed on cell surfaces through a mechanism involving glycerophosphatidylinositol anchors [1]. Although GPC3 is expressed in a variety of tissues during development, its expression is at least partially inhibited in most adult tissues by DNA methylation in the promoter region [2]. However, GPC3 protein is overexpressed in about 70% of hepatocellular carcinoma (HCC) patients [3, 4] and stimulates classic Wnt signal transduction [5] through interaction with Wnt ligand, which promotes Wnt/frizzled binding to promote HCC growth [6]. Activation of the classic Wnt signaling pathway is one of the most frequent events associated with malignant transformation of HCC [7, 8]. Based on GPC3′s ability to increase Wnt signaling, we hypothesized that overexpression of GPC3 promotes HCC growth by stimulating the classic Wnt pathway.

Therapeutic monoclonal antibody that recognizes an epitope in the C-terminal portion of GPC3 (524–563) (hGC33), which recognizes the C-terminal epitope of GPC3 (524–563), inhibits tumor growth in subcutaneous xenografts of HepG2 and Huh-7 in mice [9, 10]. hGC33 has been humanized by complementary decision-region transplantation, and its anticancer effect is as effective as hGC33 for HepG2 xenotransplantation, and hGC33 has been used in clinical trials [11]. These results suggest that hGC33 has important antitumor activity, and anti-GPC3 treatment will directly inhibit the proliferation and/or survival of HCC cells by blocking Wnt and/or other signaling pathways.

However, due to the complex pathogenesis of HCC, the efficacy of blocking GPC3 alone is limited [12]. Therefore, exploring the detailed mechanism of HCC pathogenesis and identifying promising biomarkers for diagnosis and prognosis of HCC may help provide effective therapeutic targets and improve the prognosis of patients. Anti-GPC3 antibodies have been proposed to increase the sensitivity of HCC to chemotherapeutic agents [13]. The anti-tumor activity of hGC33 in combination with standard chemotherapy drugs has been evaluated recently [14]. Sorafenib is a novel, oral targeted HCC drug that can directly inhibit the proliferation of tumor cells by blocking the cell-signaling pathway mediated by RAF/MEK/ERK to curb tumor growth [15, 16]. However, the nontargeted distribution of sorafenib in vivo and abnormally activated Wnt signals in HCC limit the drug’s effectiveness and increase its side effects [14–16]. Wnt binds to receptors in the Frizzled family to activate intracellular signal transduction that regulates cell proliferation, apoptosis, and cell migration, and cause drug resistance in many tumors, such as HCC [17–19].

In HepG2 xenotransplantation models, the combination of hGC33 and sorafenib is more effective in inhibiting tumor growth than is sorafenib alone [15], and drug delivery with polymer nanocarriers has received much attention in cancer treatment. Anti-cancer drug-loaded nanocarriers can prevent nonspecific distribution and nonspecific degradation in vivo, improve drug bioavailability and anti-tumor targeting, and simplify the evaluation of pharmacokinetics and treatment [15, 16]. In a variety of polymer-based nanoparticles, poly (lactic co glycolic acid) (PLGA)-based formulations are considered ideal and safe drug carriers [17, 18]. In this regard, the poly(ethylene glycol)-*b*-poly(d,l-lactide-co-glycolide) (PEG-*b*-PLGA) are based on polyethylene glycol and PLGA copolymers, which are safe and nontoxic after hydrolysis, and have been approved by the US Food and Drug Administration [19–22]. Therefore, intravenous injection with nanocarrier of PEG-*b*-PLGA copolymer is a promising strategy to achieve targeted delivery and enhance efficacy. Furthermore, the application of specific antibody hGC33 against GPC3 molecules on HCC cell membrane can not only improve the delivery of nanodrug targeting in vitro and in vivo [18, 19], but also block Wnt and/or other signal pathways connected with GPC3, inhibit the proliferation and/or survival of cancer cells, and may achieve synergistic anti-tumor activity.

In this study, we explored whether hGC33 antibody-modified copolymer PEG-*b*-PLGA nanoparticles can facilitate the delivery of sorafenib (hGC33-SFB-NP) delivery to HCC in vivo and in vitro, and improve the efficiency of HCC treatment through HCC-active targeting to change the drug pharmacokinetics. According to the particle size, zeta potential, particle morphology, drug entrapment efficiency, drug loading capacity, and in vitro drug release, the targeted NP was comprehensively characterized. In vitro targeting ability is characterized by cell uptake of HepG2 hepatoma cells. The biodistribution and the synergistic therapeutic effect of hGC33-SFB-NP on HCC were evaluated by comparing sorafenib and SFB-NP. Our results demonstrated that hGC33-SFB-NP can target GPC3^+^ HCC. It can inhibit cell–cycle progression, cell proliferation, and tumor invasion by inhibiting Wnt and Ras/Raf/MAPK signal pathways and synergistically inhibit the progression of liver cancer.

## Materials and Methods

### Materials

The hGC33 antibody, 3-(4,5-dimethylthiazol-2-yl)-2,5-diphenyltetrazolium bromide (MTT), 4′,6-diamidino-2-phenylindole, dihydrochloride (DAPI), 5,5′,6,6′-tetrachloro-1,1′,3,3′- tetraethyl-benzimidazolyl carbocyanine iodide (JC-1), and dimethyl sulfoxide (DMSO) were obtained from Sigma—Aldrich, Inc. (St. Louis, MO). 1-(3-dimethylaminopropyl)-3-ethyl- carbodiimide hydrochlorideand *N*-hydroxysuccinimide were obtained from Qiyun Biotech (Guangzhou, China). The bicinchoninic acid (BCA) protein quantification kit, coumarin-6, and the Annexin V-FITC/PI apoptosis detection kit were purchased from Beyotime Biotechnology (Shanghai, China). PEG-*b*-PLGA-maleimide diblock copolymer (mal-PEG-*b*-PLGA; 25,000–30,000 Da, PLGA, LA:GA, w/w; PEG, 13–15%) was purchased from Polyscitech (West Lafayette, IN, USA). PI3K antibody kit (9655#), p-Akt antibody kit (9916#), mTOR antibody kit (9964#), Bcl-2 family antibody kit (9942#), apoptosis antibody kit (9915#), and secondary goat anti-rabbit and anti-mouse antibodies were purchased from Cell Signal Technology (Danvers, MA, USA); cyclin B1 and cyclin-dependent kinase were purchased from Abcam Biological Technology (USA). Antibodies to phospho-Rb, cyclin D1, checkpoint kinase 1 (CHK1), P53, phosphorylated breast cancer susceptibility gene 1 (p-BRCA1), RAD51, cytochrome C, and matrix metalloproteinase (MMP2 and MMP9) were purchased from Abcam (USA). All other analytical grade chemicals, reagents, and solvents are obtained from standard suppliers and were used without further purification.

### Cells and Animals

HCC cell line HepG2 (obtained from the American type culture collection (Manassas, VA, USA) was cultured in the DMEM medium (Invitrogen, Carlsbad, CA, USA) supplemented with 10% FBS (HyClone, Logan, UT, USA) and 80 U/ml penicillin and 80 μg/ml streptomycin in a humidified atmosphere of 5% CO_2_ at 37 °C. BALB/C nude mice, weighing 20–22 g (5–6 weeks) were provided by Nanjing Junke Biotechnology Co., Ltd. (China). BALB/C nude mice were reared in a SPF room. All animal care and treatment were carried out according to the animal care requirements of Anhui University of Science and Technology. All experimental protocols have been reviewed and approved by animal experiment ethics committee of Anhui University of Science and Technology (Approval No.: 2019dw013).

### Preparation of NPs

To prepare NPs, mal-PEG-*b*-PLGA and SFB or coumarin-6 were weighed and dissolved in the organic phase (3:2 v/v dichloromethane/acetone). The solution was added to a polyvinyl alcohol (PVA) solution (5% w/v) drop by drop with continuous vortexing. The mixture was sonicated intermittently with a probe sonicator (output power of 550 W, 8 times) on ice to create an oil–water emulsion. The emulsion was added to a PVA (1% w/v) solution with magnetic stirring. SFB and coumarin-6 NPs were collected by centrifugation at 8000 rpm for 30 min and washed three times in Milli-Q water.

To generate hGC33-NP by thioether bonds formed by the reaction of maleimide with free sulfhydryl residues in antibody hGC33, hGC33 antibody was mixed with maleimide-functionalized NPs at a molar ratio of 5:1 (hGC33: mal-PEG-*b*-PLGA) and incubated at 4 °C for 16 h with continuous stirring. hGC33 was conjugated to the NPs through the reaction of sulfhydryl groups on the antibody hGC33 with the maleimide groups of the PEG chains. Unconjugated antibodies were removed by passage through Sepharose CL-4B columns. Efficient protein conjugation was confirmed with a BCA kit (Thermo Fisher Scientific, Waltham, MA, USA).

### Characterization of Nanoparticles (NPs)

#### Morphology, Particle Size, Encapsulation Efficiency (EE), and Stability of NPs

The morphology of NPs was evaluated with transmission electron microscopy (TEM, H-600; Hitachi, Tokyo, Japan). Drug-free hGC33-NPs and drug-free NPs were recorded by FTIR spectrophotometer (Thermo Nicolet, Madison, WI, USA) using potassium bromide. The mean particle size and zeta potential of NPs were characterized with a Malvern Zetasizer ZEN3600 Nano ZS (Malvern Instruments, Malvern, UK) at 20 °C. The drug encapsulation efficiency (EE) and drug loading content (LC) efficiency were assessed by ultrafiltration. Samples were loaded into an ultrafiltration device (100 kMWCO; Sartorius, Göettingen, Germany) and centrifuged at 8000 rpm for 25 min at 4 °C to remove free drug. The same volume of each sample was dissolved in acetonitrile to confirm the total amount of drug. The concentration was measured by high-performance liquid chromatography. The absorption wavelength was 266 nm. The following formula was used to calculate the drug EE (%) of the NPs: (weight of drug entrapped/total weight of drug) × 100%. LC (%) was calculated as (weight of encapsulated drug/weight of NPs) × 100%. To understand the stability of NPs at room temperature, the change in NP size was evaluated by dynamic light scattering (DLS) at predetermined time points (0.5, 1, 2, 4, 8, 12 h, 16 h, 20 h, and 24 h) at 25 °C.

#### In Vitro Drug Release of Drug and Cellular Uptake Assay

The drug from NPs was investigated using dialysis bags with a molecular weight cutoff of 10 kDa. Briefly, 1 mL of NPs was loaded into a dialysis bag (MWCO 8,000–10,000 Da; Spectrum Labs Inc., CA, USA). The dialysis bags were immersed in PBS and agitated with a magnetic stirrer at 25 °C. The drug-release profiles of NPs were measured in 100 mL of 0.2 M phosphate-buffered saline (PBS; pH = 7.4) for 7 days. The concentration of drug in the samples was measured with high-performance liquid chromatography. In subsequent studies, hGC33-coumarin 6-NP having the same particle size as hGC33-SFB-NP was used to evaluate the targeting of hGC33-SFB-NP. HepG2 (GPC3^+^), and Li-7 (GPC3^−^) were incubated with hGC33-coumarin 6-NP for 0.5, 2, or 4 h at 37 °C in 5% CO_2,_ respectively. The co-cultured cells were washed and fixed with 4% formaldehyde for 10 min; the cell nuclei were stained with 5 μg/mL Hoechst 33,342 for 15 min to locate intracellular NPs. Confocal microscopy to analyze intracellular nanoparticle images was analyzed with confocal microscopy (Olympus FV1000; Olympus Corporation, Tokyo, Japan).

#### In Vitro Cellular Effect

The cytotoxicity of free hGC33(Ab), free SFB, hGC33-null-NP, or hGC33-SFB-NP was determined using an MTT assay. HepG2(GPC3^+^) cells and Li-7 (GPC3^−^) cells in logarithmic phase were seeded into a 96-well plate at a density of 4000 cells per well followed by incubation for 48 h at 37 °C in 5% CO_2_. The cells were treated with hGC33, free SFB, hGC33-null-NP, or hGC33-SFB-NP for 48 h at 37 °C in 5% CO_2_. After co-cultivation for a definite time, anti-cell proliferation activity was determined by MTT assay as described [20]. The absorbance of each well was measured at 490 nm and calculated half of the maximum inhibitory concentration value (IC 50) with SPSS 17.0.

### Measurement of Cell Invasion Ability

Cells in logarithmic growth phase were seeded onto 6-well plates at a density of 5 × 10^4^ cells/well, scratched with a suction head, and replaced with serum-free culture medium. Wound healing was recorded at 0 h, 24 h, and 48 h in the control group and experimental group. At the same time, the same number of cells was inoculated into Transwell chambers and treated with free hGC33, free SFB, hGC33-null-NP, or hGC33-SFB-NP. Five hundred microliters of 10% FBS medium was added into the lower chamber. After incubation for 24 h, the Transwell chamber was taken out, and cells were fixed with 4% paraformaldehyde, and stained with 0.1% crystal violet. The size of wound healing and the number of migration cells were calculated to evaluate the migration ability.

### Cell Cycle Determination

After incubatation overnight in 6-well plates, the cells were treated with free hGC33 (Ab), free SFB, hGC33-null-NP, or hGC33-SFB-NP for 24 h, then collected and fixed with ethanol. After staining with propidium iodide, flow cytometry was performed, and the cell cycle was analyzed with modifit 3.0 (Verity Software House, Topsham, ME).

### Western Blotting

To evaluate the activation status of signal pathway and the expression of target molecules, the cells were incubated overnight in 6-well plates, and free hGC33, free SFB, hGC33-null-NP, or hGC33-SFB-NP were applied for 24 h. The cells of each treatment group were collected, and protein was extracted and measured. The protein concentration was measured and calibrated with BCA protein kit (Biosharp, Hefei, China). Protein of the samples was separated by twelve alkyl sulfate polyacrylamide gel electrophoresis, transferred to a PVDF membrane, and sealed with fat-free milk. The first antibody (diluted 1:1000) was incubated overnight at 4 C, and the second antibodies (1:2000) were incubated for 1 h at room temperature. The bands were visualized with ECL substrates (Thermo Fisher Scientific Waltham, MA, USA), and the images were displayed by gel image analysis system, and β-actin was used as control.

### In Vivo Anti-Tumor Activity

The inhibition of free hGC33, free SFB, hGC33-null-NP, SFB-NP, and hGC33-SFB-NP on the growth of HCC in vivo was determined. According to regulations and guidelines on animal health of the ethics committee of Anhui University of Technology, all experiments were conducted in BALB/c mice in cages in a temperature-control room (23 ± 2 °C) with 12 h/12 h light/dark cycle. A 50-μl suspension containing 5 × 10^6^ live HepG2 cells was injected subcutaneously into the right abdomen of 5-week-old female BALB/c mice (20–22 g). When the tumor volume reached about 50 mm^3^, the mice were randomly divided into 6 groups (10 mice in each group). Normal saline control NS (200 mg/kg null NP in 200 μL PBS), hGC33-null-NP (hGC33-null-NP in 200 μL PBS, equivalent to hgc33 = 100 μg/kg/time), free hGC33 (hGC33 in 200 μL PBS, 100 μg/kg/time), free SFB (SFB dose: 8 mg/kg/time), SFB-NP (SFB dose: 8 mg/kg/time), and hGC33-SFB-NP (equivalent to SFB = 8 mg/kg/time, hGC33 = 100 μg/kg/time) were injected via tail vein every 2 days for 10 times. The weight and tumor size of mice were measured every four days. The calculation formula of tumor volume was volume = 0.5 × *L* × *W*^2^, where L and W represent the length and width of tumor, respectively. Four weeks after administration, the animals were anesthetized with diethyl ether, and the tumor size and weight were measured. In addition, tumor, heart, liver, kidney, lung, and spleen were removed, fixed with 4% paraformaldehyde solution, embedded in paraffin, sectioned, and stained with hematoxylin and eosin to evaluate the histological changes by digital microscopy.

### Statistical Analysis

Data are presented as the mean ± standard deviation (SD) and were evaluated by analysis of variance with SPSS 18.0. Pairwise statistical comparisons were performed using a two-tailed Student’s t test. Differences were considered statistically significant for *P* < 0.05.

## Results

### *Characterization of NPs and Drug Release *In Vitro

**Fig. 1:**
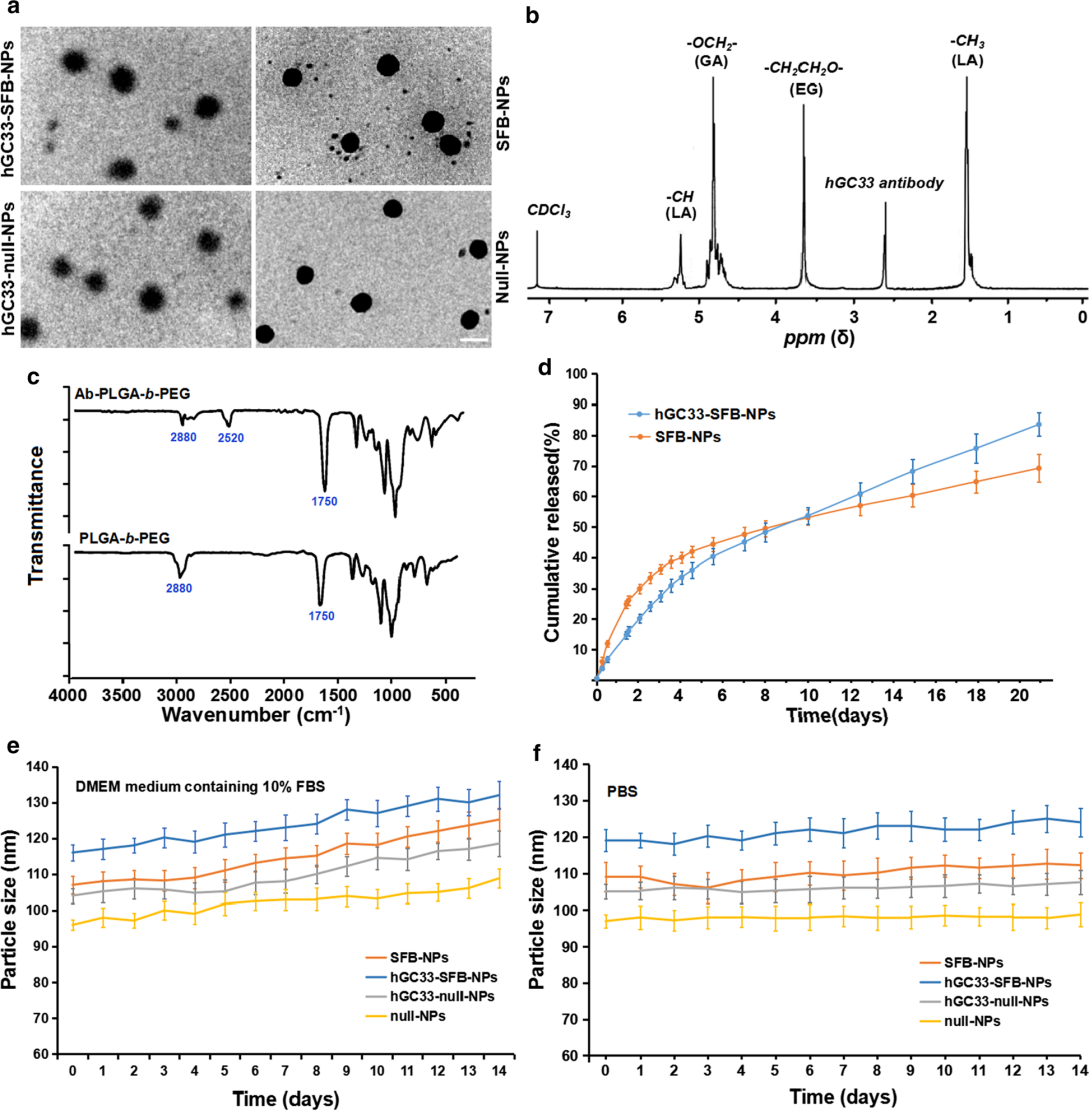
Characterization of NPs. **a** TEM characterization of NPs, the scale bar indicates 100 nM; **b** the ^1^H NMR spectra of synthetic hGC33-PLGA-*b*-PEG in CDCl3; **c** FTIR spectra of hGC33-PEG-*b*-PLGA and PEG-*b*- PLGA; **d** cumulative release profiles of SFB-NP and hGC33-SFB-NP in PBS (pH = 7.4) at 37 °C; **e** size changes of NPs incubated in DMEM medium containing 10% FBS over 14 d; **f** size changes of NPs incubated in PBS over 14 d. SFB, sorafenib; TEM, transmission electron microscopy; NPs, nanoparticles; ^1^H NMR, ^1^H-nuclear magnetic resonance spectroscopy; FTIR, Fourier transform infrared spectroscopy

**Fig. 2:**
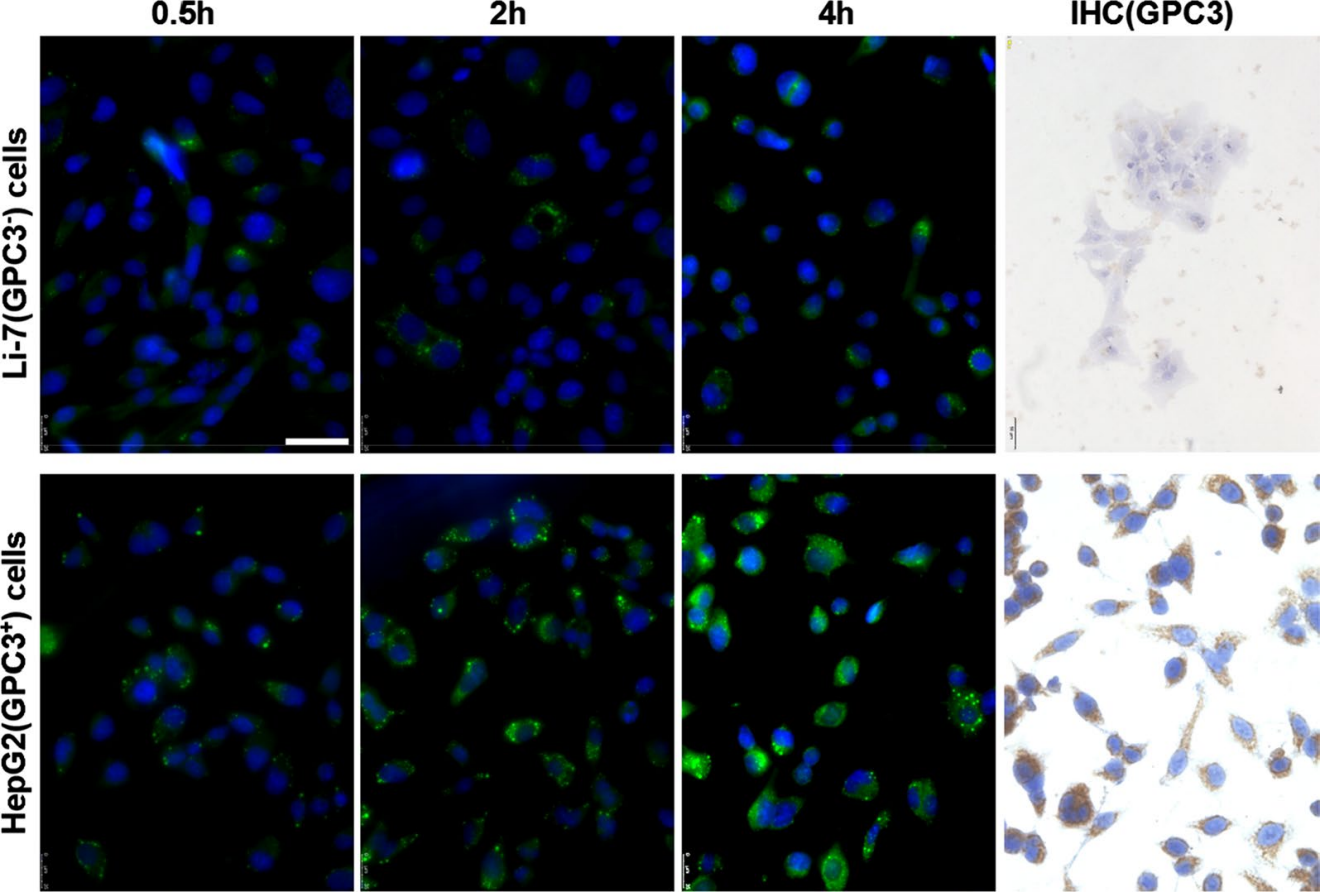
Expression of GPC3 and uptake of hGC33-coumarin6-NP in Li-7 and HepG2 cells. The cells, inoculated in the culture plate, were washed with PBS and incubated with 100 μg/ml hGC33-coumarin6-NP in DMEM for 2, 4, and 8 h. Nuclei were stained with DAPI, and the cells were fixed and detected by confocal laser scanning microscopy. GPC3 was not detected in Li-7 cells, but was highly expressed in HepG2 cells as detected by immunocytochemistry. The scale bar indicates 50 μM

Because the diameter and surface properties of NP affect cell uptake, drug release, and NP distribution in vivo, we characterized the prepared NP with corresponding parameters. The morphology, particle size, and particle size distribution of SFB-NP, hGC33-null-NP, and hGC33-SFB-NP polymers are summarized in Fig. 1. The morphology of hGC33-SFB-NP observed by transmission electron microscope (Fig. 1a) shows rigid nuclei with fuzzy edges, which indicates that hGC33 is present on the surface of NPs. The particle sizes of SFB NP, hGC33-null-NP, and hGC33-SFB-NP ranged from 100 to 150 nm and had typical unimodal particle size distribution. The average diameter of hGC33-SFB-NP (120.2 ± 10.2 nm) was slightly larger than that of hGC33-null-NP, SFB-NP, and null NP (Fig. 1a, Table 1). Thin spherical unclear films with a single surface were present on the surface of hGC33-SFB-NP and hGC33-null-NP, which indicated that antibody hGC33 was present on the surface of NPs. The increased size for hGC33-SFB-NP and hGC33-null-NP confirmed the existence of hGC33 film. The synthesis of hGC33-SFB-NP was confirmed with ^1^H-NMR (Fig. 1b) before NP preparation. The peaks at 5.2 ppm and 1.58 ppm were assigned to -CH3 protons of lactic acid; the peak at 4.8 ppm was assigned to -OCH2- of glycolic acid; and the peak at 3.6–3.8 ppm was assigned to the -CH2CH2O- protons of PEG repeat units. Surface chemistry of PEG-*b*-PLGA and Ab-PEG-*b*-PLGA NPs was also studied by FTIR spectroscopy (Fig. 2c). In the spectrum of PEG-*b*-PLGA polymer, a strong band at about 1750 cm^−1^ originated from the stretch of the carbonyl groups (C = O) in the PLGA chain. A band at 2880 cm^−1^ was due to stretching of an a –CH group in the PEG chain. At the same time, a peak appeared at 2520 cm^−1^ that we attributed to the -SH stretching peak of the hGC33 antibody. The ^1^H NMR and FTIR results indicated that antibody was grafted on the backbone of the PEG-*b*-PLGA polymers.

**Table 1:** Physical and chemical characteristics of NPs

Co-polymer	Particle size (nm)	PDI	Zeta potential (mV)	LC(%)	EE(%)
hGC33-SFB-NP	120.2 ± 8.2	0.23 ± 0.14	− 18.2 ± 2.2	4.8 ± 0.5	68.5 ± 6.7
SFB-NP	109 ± 7.1	0.19 ± 0.12	− 15.9 ± 2.1	5.3 ± 0.6	85.7 ± 4.3
hGC33-null-NP	105.5 ± 8.4	0.21 ± 0.11	− 18.5 ± 1.8		
Null-NP	98.1 ± 6.9	0.18 ± 0.11	− 15.1 ± 2.5		

Values are reported as mean ± SEM (*n* = 3). NP, PEG-*b*-PLGA nanoparticle; PDI, polydispersity index; EE%, encapsulation efficiency %; LC%, drug loading %

Interestingly, hGC33-SFB-NP and SFB-NP nanoparticles in DMEM medium containing 10% FBS (pH = 7.4) had rapid drug release until about 4 days, then relatively slow and stable drug release; the cumulative SFB release of hGC33-SFB-NP and SFB-NP over 20 days was about 77% and 65%, respectively (Fig. 1d). The difference may be due to the hydrophilic molecule on the surface of the PEG-*b*-PLGA matrix, which may hasten degradation of the nanoparticles by increasing hydration and thereby promoting hydrolysis. To determine the stability of the prepared NPs, the hGC33-SFB-NP, hGC33-null NP, null-NP, and SFB-NP were placed in DMEM medium containing 10% FBS (pH = 7.4) and in PBS (pH = 7.4). The sizes of the various NPs remained stable for more than 2 weeks. SFB was released from hGC33-SFB-NP in a sustained and stable manner for 14 days, but there was a modest change in the size of the particles in DMEM medium compared with that in 10% FBS (Fig. 1e, f). Stability of hGC33-SFB-NP would be appropriate for SFB having a sustained biological role.

Large zeta potential can cause strong electrostatic repulsive interaction between NP and maintain the stability of the NP dispersion system [23, 24]. As shown in Table 1, the zeta potentials of hGC33-SFB-NP, hGC33-null-NP, and SFB-NP are − 18.2 ± 2.2 mv, − 18.5 ± 1.8 mv, and − 15.9 ± 2.1 mv, respectively, which may be caused by the negative charges generated by the aldehyde group, carboxyl group, and phosphate group in the antibody hGC33 glycoprotein. The negative charged NPs can lead to the high stability of NP suspension. In addition, because the surface of cells is negatively charged in normal physiological environments, the prepared NPs repel cells with low charge and are less toxic to tissues and cells. In addition, the size distribution of null-NP and SFB-NP (polydispersity index [PDI] 0.18 and 0.19, respectively) was slightly but not significantly smaller than that of hGC33-null-NP (PDI = 0.21) and hGC33-SFB-NP (PDI = 0.23).Thus, the size of the nanoparticles has good uniformity. The particle size, particle size distribution, and zeta potential of NP are shown in Table 1 according to the results of SFB encapsulation efficiency and loading content. The unconjugated GPC3 antibody hGC33 was removed by ultracentrifugation, and the binding efficiency of hGC33 antibody to NPs was evaluated. BCA protein analysis showed that the binding efficiency of hGC33 antibody to NPs was 79.5% ± 2.9%.

### *hGC33-Coumarin 6-NP Effectively Targets GPC3*^+^*HCC Cell Line HepG2 Cells*

To find out whether the hGC33 antibody of nanoparticles still has the ability to specifically target GPC3, we used GPC3^+^ HepG2 and GPC3^−^ Li-7 cells as target cells and hGC33-coumarin 6-NP as tracer nanoparticles, and incubated the different cells for 2 h, 4 h, and 8 h. The cells were washed with PBS 3 times and reacted with DAPI to stain the nucleus. The cells were fixed and photographed the with Leica fluorescence microscope (DMi8, Germany). It was found that the green fluorescence in HepG2 cells was significantly higher than that in Li-7 cells at the same incubation time (Fig. 2), indicating that the amount of hGC33-coumarin 6-NP entering HepG2 cells was significantly higher than that in Li-7 cells. The results documented that the hGC33 antibody on hGC33-Coumarin6-NP still had the ability to target GPC3 and mediate the internalization of nanoparticles. The expression of GPC3 in HepG2 cells and Li-7 cells was examined with indirect fluorescence and cytochemical staining. The results showed that HepG2 cells overexpressed GPC3, while Li-7 cells did not express GPC3 (Fig. 2).

### hGC33-Null-NP Inhibits Proliferation of HepG2 Cells

**Fig. 3:**
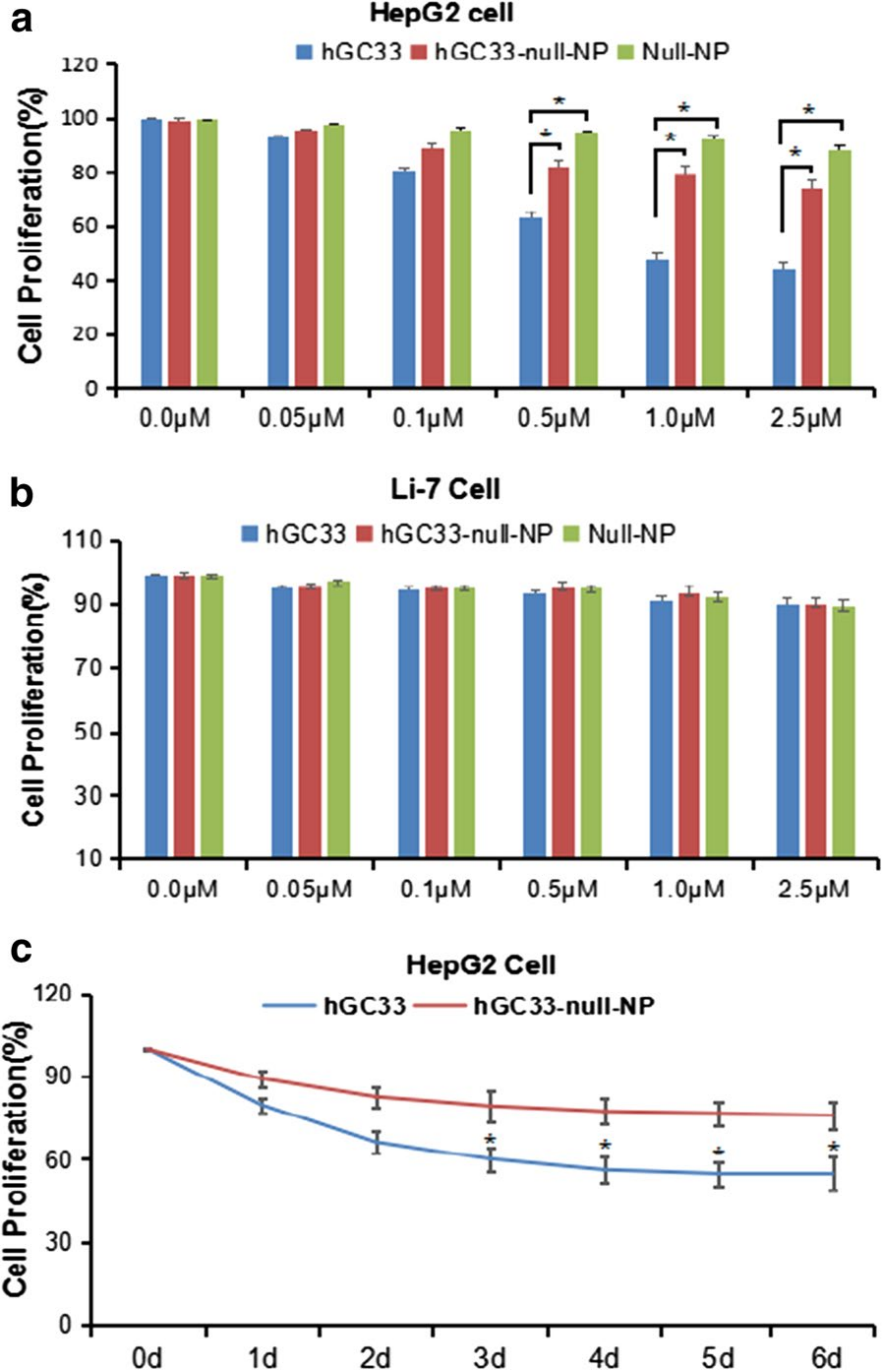
The proliferation of HepG2 and Li-7 cells was inhibited by hGC33, null-NP, and hGC33-null-NP. **a** Cell proliferation test was performed on GPC3-positive HepG2 cells treated with hGC33, null-NP or hGC33-null-NP; **b** cell proliferation tests were conducted on GPC3 negative Li-7 cells treated with hGC33, null-NP, and hGC33-null-NP. The cells were incubated with 0–2.5 μM hGC33, null-NP, or hGC33-null-NP for 1 day. The proliferation of the cells was measured with MTT method and standardized as untreated cells. All values represent the mean ± SD. Compared with the control group (0 μM) without antibody treatment, *P < 0.01; **c** the representative results of response of HepG2 to hGC33, null-NP, and hGC33-null-NP in hGC33 treatment (1.0 μM)

To determine whether hGC33-modified NP (hGC33-null-NP) could inhibit the growth of HCC, we measured the cell growth inhibition of GPC3 positive HCC cell line HepG2 and GPC3 negative cell line Li-7. We found that both hGC33-null-NP and hGC33 inhibited the growth of the GPC3-positive HCC cell line HepG2 after 24 h of treatment, but hGC33 had more significant inhibitory effect on HepG2 cells than hGC33-null-NP (Fig. 3a); in contrast, hGC33-null-NP and hGC33 did not affect the growth of the GPC3-negative Li-7 cell line (Fig. 3b). The representative time course of hGC33-null-NP (equivalent to 0.1 μ m hgc33) and hGC33 (0.1 μ m) on HepG2 cell proliferation is illustrated in Fig. 3c. Because hGC33 is a C-terminal peptide that recognizes GPC3, hGC33 is superior to hGC33-null-NP in inhibiting HepG2. The results suggest that the hGC33 molecule in hGC33-null-NP may have space blocking effect, which may affect the binding ability of hGC33 to epitopes.

### hGC33-Null-NP Inhibits Cell Cycle of HepG2 Cells

**Fig. 4:**
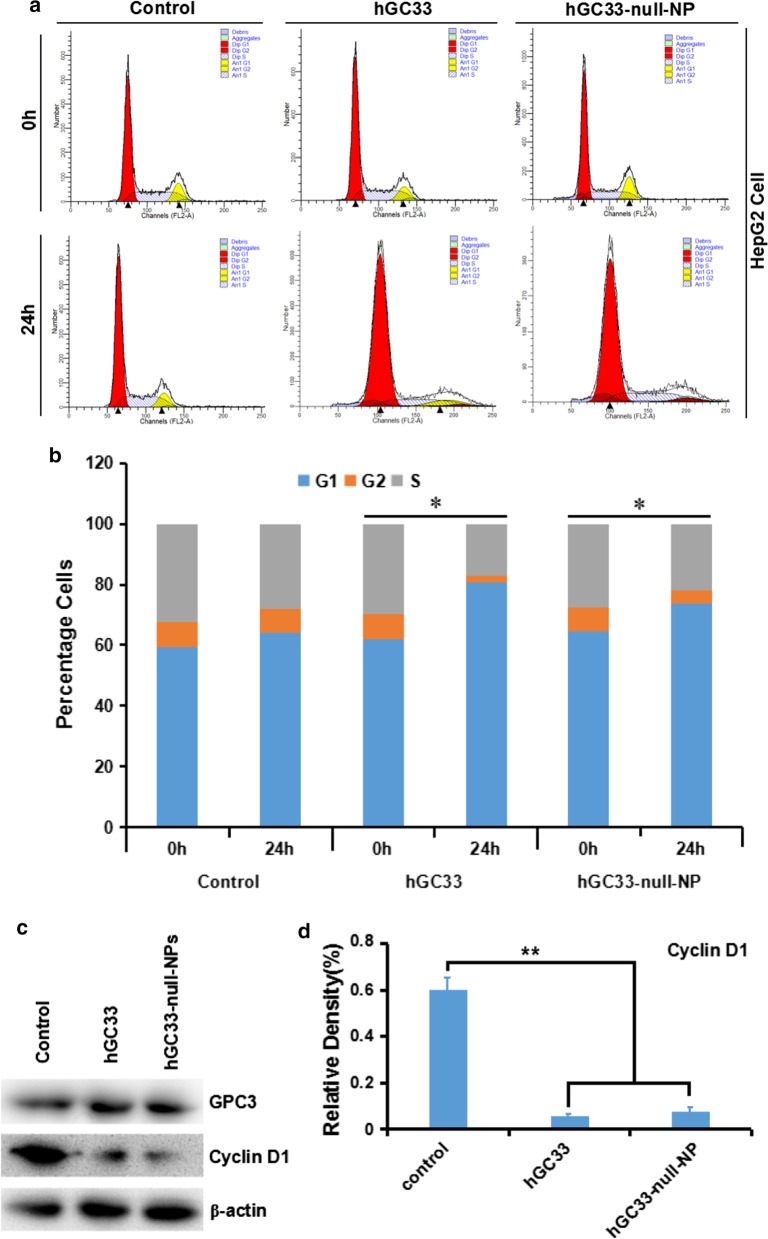
hGC33-null-NP and hGC33-induced cell cycle arrest in G1 phase and inhibited cyclinD1 expression in HepG2 cells. **a** Representative cell–cycle diagram of various groups treated with hGC33-null-NP and hGC33. **b** Cell cycle analysis of various groups treated with hGC33-null-NP and hGC33. The HCC cells were incubated with 0.5 μm hGC33 or hGC33-null-NP (with the molar concentration of hGC33 as reference). *P < 0.05, the G1 phase of hGC33-null-NP or hGC33 cells was compared with that of 0 h cells. **c**, **d** After hGC33-null-NP or hGC33 treatment, cyclinD1 was significantly down regulated in HepG2 cells compared with that in the control group

To understand the mechanism of the molecular activity of hGC33 antibody modified on hGC33-null-NP nanoparticles, we studied the cell–cycle progression of HepG2 treated with hGC33-null-NP. In the HepG2 cell line, hGC33-null-NP and hGC33 significantly increased the proportion of cells in G1 (Fig. 4a, b), indicating that both hGC33-null-NP and hGC33 could induce cell cycle arrest in G1 phase. Moreover, hGC33-null-NP and hGC33 could significantly down regulated cyclinD1 expression in HepG2 cells (Fig. 4c, d).

### Wnt Activation in HepG2, Huh-7 and Li-7 Cells

**Fig. 5:**
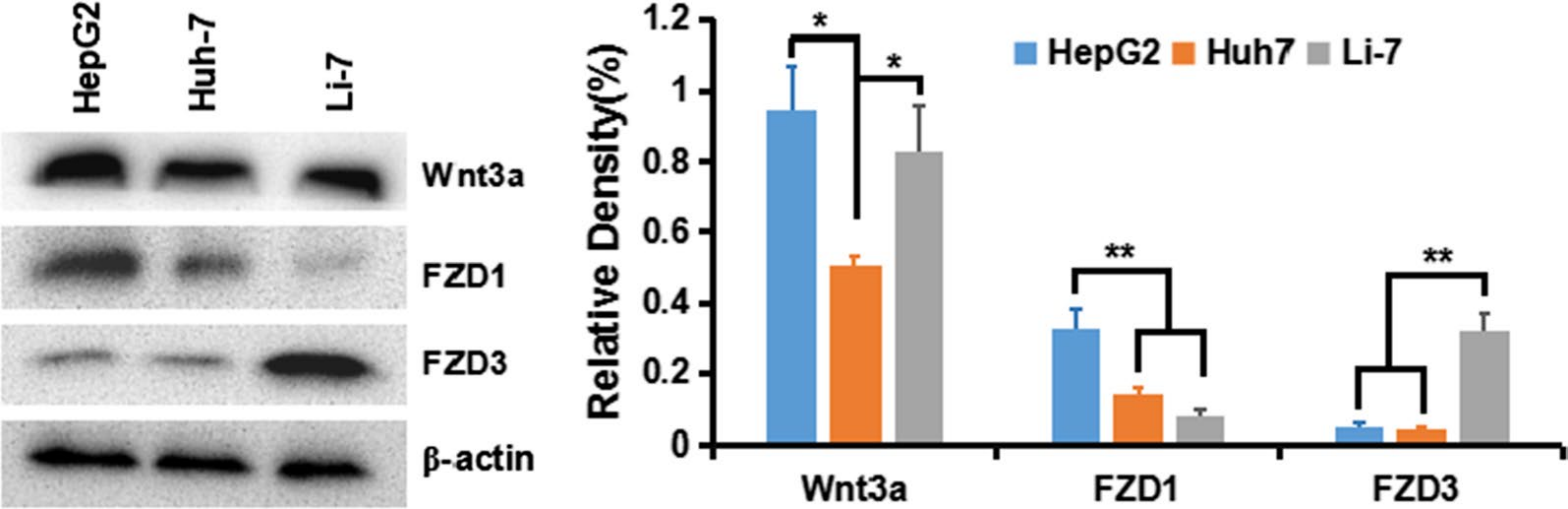
Expression of Wnt3a, FZD1, and FZD3 in HCC cell lines

To understand activation of the classical Wnt/β-Catenin signal in HCC cells, we first detected the expression of Wnt ligand and receptor crimp protein (frizzled, FZD or Frz) in various HCC cell lines: HepG2 (GPC3^++^), Huh-7 (GPC^+^), and Li-7 (GPC3^−^). The results showed that Wnt3a and FZD receptor of transduce the β-Catenin pathway were expressed in all cell lines, especially in HepG2 and Huh-7 cells (Fig. 5).

### hGC33-Null-NP Inhibits Wnt Signal Transduction-Dependent Cell Proliferation Induced by Wnt3a

**Fig. 6:**
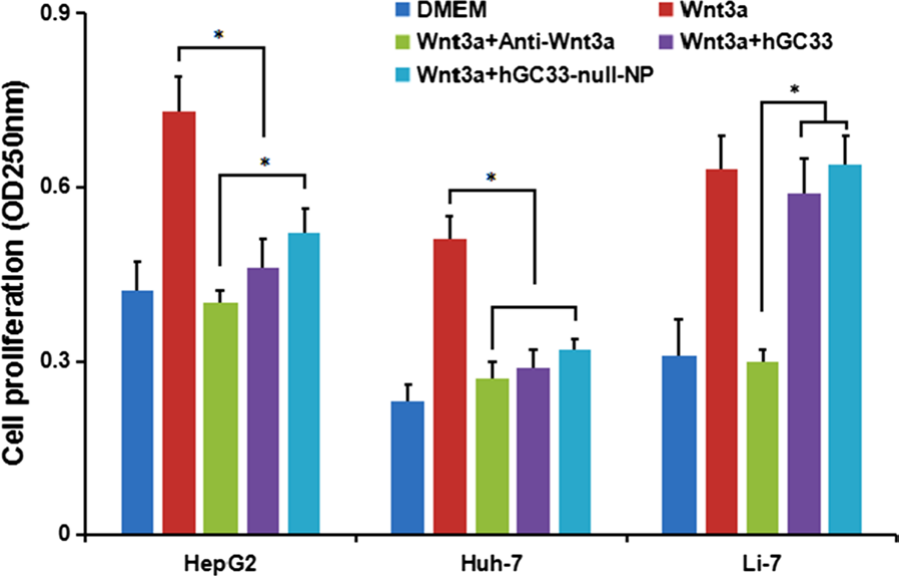
GPC3-activated Wnt signal transduction in HCC. Fifty percent Wnt3a DMEM medium was added with anti-wnt3a antibody, hGC33, or hGC33-null-NP. HepG2 (GPC3^++^), Huh-7 (GPC3^+^) and Li-7 (GPC3^−^) cells were co-incubated for 48 h, and cell proliferation was measured by MTT assay. The data were expressed as mean ± SD (*P < 0.01)

Some studies have shown that the extracellular part of GPC3 may be a co receptor of Wnt, which promotes Wnt/β-Catenin signal activation and transduction. Therefore, when HepG2 (GPC3^+^), Huh-7 (GPC3^+^), and Li-7 (GPC3^−^) cells were co-incubated with hGC33 and hGC33-null-NP, the activation of Wnt/β-catenin signal was blocked by hGC33 and hGC33-null-NP, and proliferation of HepG2 and Huh-7, but not Li-7, in Wnt3a-conditioned medium was inhibited. That proliferation of Li-7 cells most likely is due to the absence of GPC3 on the surface of Li-7 cells (Fig. 6). Our results indicate hGC33 and hGC33-null-NP nanoparticles specifically bind to GPC3 molecules on cell membrane. hGC33 and hGC33-null-NP partially neutralize the mitogenic activity of Wnt3a and inhibit the Wnt/β-catenin signaling pathway. However, the inhibition of proliferation by hGC33-null-NP nanoparticles was less than that of hGC33. Perhaps the spatial structure of the nanoparticles interferes with hGC33-null-NP and limits the function of the hGC33 molecule on the nanoparticles so they cannot completely block the interaction between GPC3 and Wnt3a.

### hGC33-Null-NP Inhibits Wnt3a-Induced Signal Transduction in HepG2 and Huh-7 Cells

**Fig. 7:**
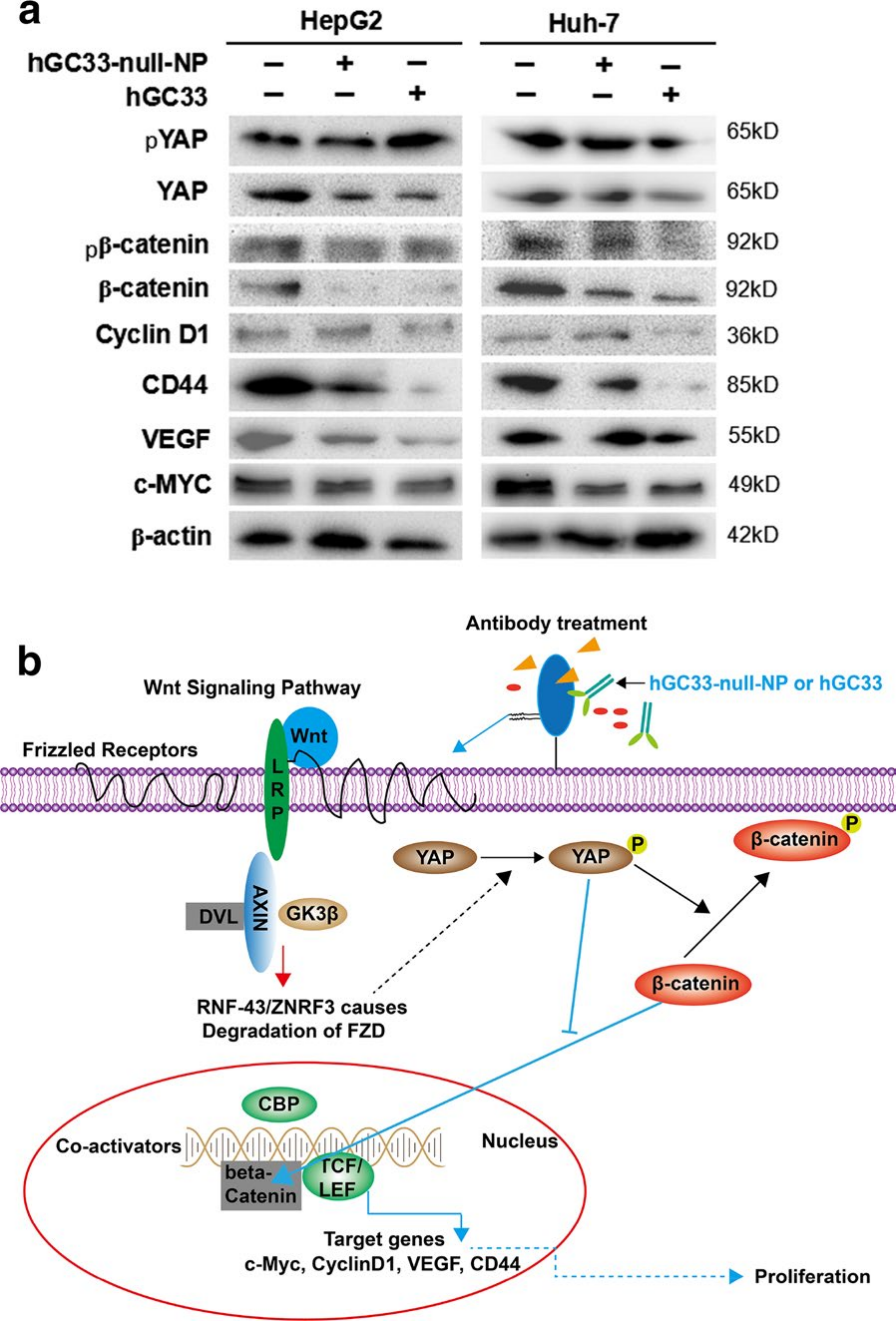
Inhibition of Wnt3a-induced β-catenin signaling by hGC33-null-NP or hGC33. **a** Compared with the control group, Wnt/β-catenin signaling pathway in HepG2 and Huh-7 cells treated with hGC33-null-NP or hGC33 was inhibited, and the levels of β-catenin and YAP were decreased, while the increased phosphorylated β-catenin and phosphorylated YAP molecules were unstable, and degraded in cytoplasm. The decreased β-catenin was difficult to maintain in the nucleus and drive the expression of CyclinD1, CD44, VEGF, and c-MYC, which resulted in the decrease of cyclinD1, CD44, VEGF, and c-myc protein levels. **b** The mechanism pattern of Wnt/β-catenin signaling pathway inhibited by hGC33-null-NP or hGC33

To understand the effect of hGC33-null-NP on Wnt/β-catenin signaling in HCC cells, we extracted the proteins of HepG2 and Huh-7 cells treated with hGC33 or hGC33-null-NP. The results of western blot showed that after hGC33-null-NP treatment, the levels of pYAP and pβ-catenin were increased, but the levels of YAP and β-catenin were decreased. Furthermore, the levels of cyclinD1, CD44, VEGF, and c-MYC in the hGC33-null-NP group were lower than those in the control group, but the level was less than with hGC33 treatment. Similar effects were observed in HepG2 and Huh-7 cells, as shown in Fig. 7.

### hGC33-SFB-NP or hGC33 Attenuates HCC Cell Migration by Inhibiting Epithelial Mesenchymal Transition (EMT)

HCC is one of the deadliest cancers in the world, and its incidence is steadily increasing. Sorafenib is the only approved standard treatment for patients with advanced HCC, as it has been shown to improve the survival rate of these patients. However, clinical and preclinical observations suggest that sorafenib therapy has limited efficacy due to the rapid development of drug resistance. Therefore, elucidating the mechanism of escape resistance to sorafenib is a major emphasis in HCC research. In recent years, more and more attention has been paid to the role of epithelial mesenchymal transition (EMT) in the progress of HCC and the development of drug resistance. EMT refers to the transformation of epithelial to mesenchymal cells, which endows cells with the ability metastasize and invade, including acquisition of stem cell characteristics, reducing apoptosis and aging, promoting immunosuppression, and participating in the occurrence and development of cancer. The loss of E-cadherin expression is considered a key step in the carcinogenesis and EMT. EMT is a developmental multi-step molecular and cellular reprogramming process that cancer cells use to achieve aggression. This is mainly through down regulating the expression of E-cadherin, keratin, mucin, ZO-1 (tight junction protein); up regulating the expression of vimentin, alpha-smooth muscle actin (α-SMA), FN fibronectin, MMPs (degradation matrix), N-cadherin, snail, slug, twist, Rho, TGF-β, FGF, type I collagen, and type II collagen to achieve the invasion, metastasis, and anti-apoptosis of EMT characteristic tumor. The changes of these protein expressions mainly involve the activation of Wnt/β-catenin and Ras/Raf/MAPK signaling pathways.

**Fig. 8:**
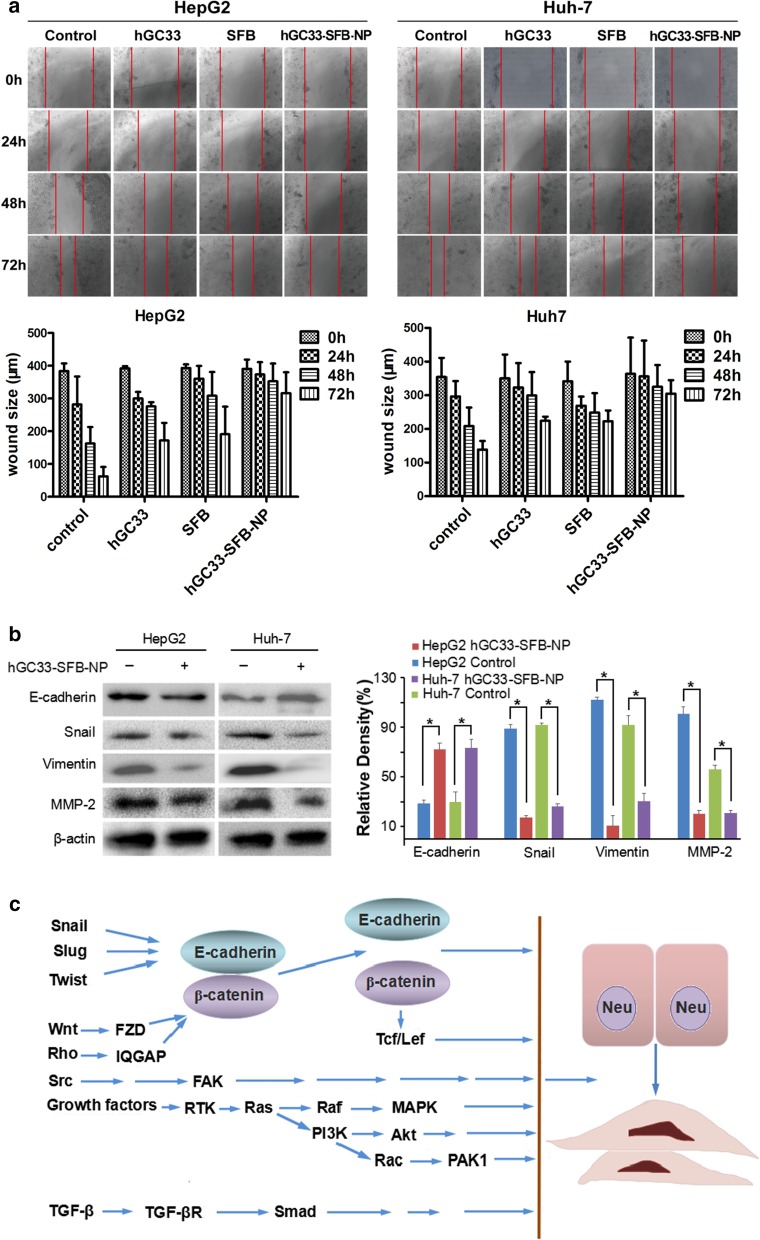
Effect of hGC33-SFB-NP on EMT inhibition. **a** Compared with the control group, the hGC33-SFB-NP treatment group had less cell migration. Photographs were taken under an optical microscope (magnification, × 200). The error value represents the standard deviation of three independent experiments. *Compared with the control group, *p* < 0.01. **b** Compared with the control group, the EMT-related proteins snail, vimentin, and MMP-2 in HCCs treated with hGC33-SFB-NP decreased, whereas E-cadherin increased. **c** Molecular mechanism of EMT. EMT, epithelial–mesenchymal transition; MMP-2, matrix metalloproteinase-2; SFB, sorafenib; NP, nanoparticle

Our experiments have shown that hGC33 antibody on the surface of NP vector can inhibit Wnt3a-induced β-catenin signal transduction, and then down regulate the expression of β—catenin, CD44, vascular endothelial growth factor (VEGF), cyclin D1, and c-MYC. Furthermore, hGC33-SFB-NP inhibits the activation of Ras/Raf/MAPK signal pathway and inhibits proliferation and apoptosis of HCC cells. hGC33 and SFB have a synergistic effect, inhibiting EMT and decreasing the migration of HCC cells (Fig. 8).

### *hGC33-SFB-NP Inhibits the Growth of Hepatocellular Carcinoma *In Vivo* and Improves the Survival Rate of Tumor-Bearing Mice*

**Fig. 9:**
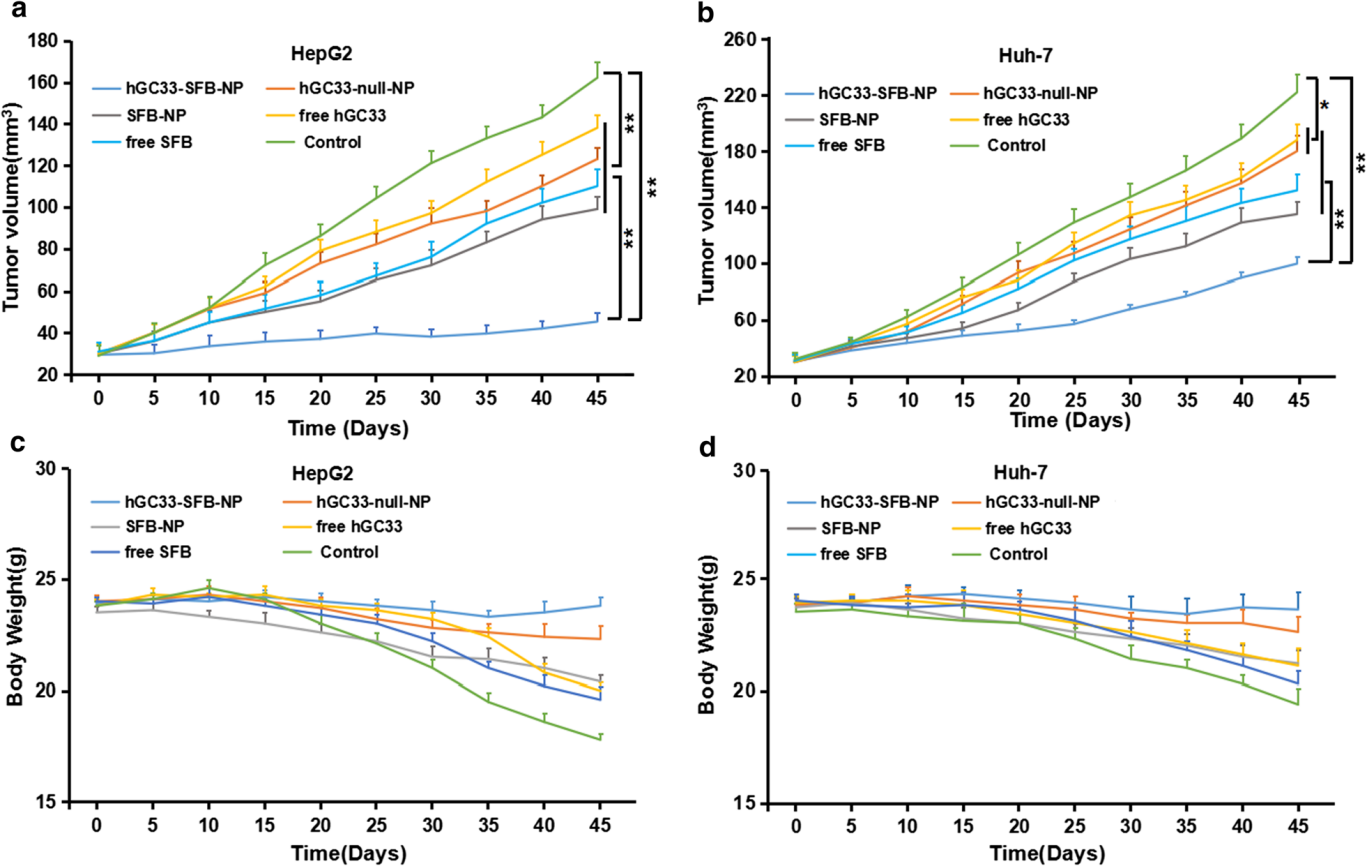
The effect of hGC33-SFB-NP on xenotransplantation of HCC in nude mice and the changes of mice weight. Liver cancer cells were inoculated subcutaneously on the back of each nude mouse (*n* = 10). After 10 days, the tumor bearing mice were treated with PBS (control), free hGC33, free SFB, hGC33-null-NP, SFB-NP, and hGC33-SFB-NP. Tumor size (**a**, **b**) and body weight (**c**, **d**) of mice were monitored at designated time points

To evaluate the anti-tumor activity of hGC33-SFB-NP in vivo, HepG2 and Huh-7 cells were inoculated subcutaneously into the right abdomen and dorsal side of female BALB/c nude mice, respectively. When the tumor xenograft growth reached about 30 mm^3^, the mice were randomly divided into groups to further evaluate the inhibition of each group (hGC33-SFB-NP, hGC33-null-NP, SFB-NP, free hGC33, free SFB, and control group) HCC effect (*n* = 5 per group). It can be seen from Fig. 9a, b that hGC33-SFB-NP significantly slowed tumor growth in mice compared with the PBS control and other treatments. Compared with the PBS control, hGC33-null-NP, SFB-NP, free hGC33, and free SFB also had some inhibition of HCC, which is because free hGC33 and free SFB directly inhibit Wnt signal and Ras/Raf/MAPK, respectively. Such pathways can inhibit the proliferation of HCC cells to a certain extent. Although the nanoparticle-modified hGC33 (hGC33-null-NP) is connected to the nanosurface through chemical bonds, it did not affect hGC33′s targeting of GPC3 molecules and inhibition of Wnt activity. Nanoparticle-loaded SFB (SFB-NP), after being endocytosed by cells, was degraded to release SFB from the copolymer to inhibit the growth of HCC. In all, the inhibitory effect of hGC33-SFB-NP on HepG2 cell grafts was, as expected, more than on Huh-7 cell grafts, probably because HepG2 expresses GPC3 molecules.

The body weight of nude mice in each treatment group also was measured, as shown in Fig. 9c, d. The body weight of the control group decreased gradually. The weight of mice in free hGC33, free SFB, SFB-NP, and hGC33-null-NP treatment groups also decreased progressively and not significantly less than in the control group. However, the weight of nude mice bearing HepG2 and Huh-7 treated with hGC33-SFB-NP only slightly decreased, and the weight remained relatively stable during the treatment cycle. These results support that the novel hGC33-SFB-NP nanodrug has no significant toxicity in nude mice, and the SFB loaded on the nanocarrier and the surface modified hGC33 can produce additive or even synergistic anti-tumor effect.

## Discussion

To examine the suitability of hGC33-SFB-NP for targeted HCC therapy, we tested the model conjugates for their ability to bind to human glypican-3 on HCC cells in vitro; to inhibit glypican-3-positive HCC cell proliferation, migration, and Wnt/β-catenin signal transduction; and inhibit HCC that overexpress glypican-3 in vivo.

To covalently bind GPC3-specific antibody hGC33 with mal-PEG-*b*-PLGA nanoparticles, we cross-linked the free sulfhydryl group in the Fc segment of hGC33 with maleimide functionalized PEG-*b*-PLGA (mal-PEG-*b*-PLGA) by forming stable thioether bonds. Conjugation is a prerequisite for targeting of GPC3-positive HCC. A series of experiments, including the changes of nanoparticle diameter and zeta potential detected by lens and the intracellular uptake of hGC33-SFB-NP, verified the targeting of hGC33-SFB-NP to HepG2 (GPC3^+^) cells. These results indicated that the binding activity of antibody hGC33 was not altered by the conjugation.

We directly detected the phagocytic effect of GPC3^+^ HepG2 and GPC3^−^ Li-7 cells on PEG-*b*-PLGA NP surface-modified hGC33 by confocal microscopy. After HepG2 and Li-7 cells were co-cultured with hGC33-coumarin 6-NP, the green signal intensity in HepG2 cells was significantly higher than in Li-7 cells, indicating that there were more nanoparticles in the HepG2 cells. This finding is consistent with the hGC33 antibody modified on the surface of PEG-*b*-PLGA NP specifically binding to glypican-3 on the surface of HCC cells and being internalized. The efficiency of hGC33-modified NP internalization depends on the expression level of GPC3 antigen on the cell surface.

We used the standard MTT assay to measure the efficiency of inhibiting the proliferation of hepatoma cells. Both hGC33-null-NP and hGC33 inhibited the growth of the GPC3-positive HCC cell line HepG2, but hGC33-null-NP and hGC33 did not affect the proliferation of GPC3-negative Li-7 cells (Fig. 3b). At the animal level, hGC33-null-NP or hGC33 alone inhibited the growth of Huh-7 and HepG2 xenografts to a certain extent, while hGC33-SFB-NP caused growth arrest of Huh-7 and HepG2 hepatoma xenografts in mice. The finding that hGC33-null-NP significantly inhibited GPC3-positive hepatoma cells showed that the inhibitory effect of PEG-*b*-PLGA NP surface-modified hGC33 on HCC cell proliferation depends on the expression of GPC3 antigen on the cell surface.

GPC3 regulates many pathways in HCC pathogenesis, including Wnt and YAP signaling [25–27]. GPC3 interacts with Wnt ligand and may be a coreceptor for Wnt and facilitate Wnt/Frizzled binding for HCC growth [28, 29]. We further examined the effect of nanodrug surface-modified hGC33 on Wnt signaling pathway in hepatoma cells. Like free hGC33, nanodrug surface-modified hGC33 inhibited the proliferation of hepatoma cells not only by blocking Wnt-induced signal transduction in HepG2 and Huh-7 cells of expressing GPC3, but also by inhibiting Wnt3a-induced β-catenin and YAP signal transduction. Previous studies have shown that YAP expression is regulated by β-catenin at the transcriptional level of HCC [30, 31]. In this study, free hGC33 and nanodrug surface-modified hGC33 inhibited Wnt3a-induced YAP activity, indicating that Yap/TAZ released from β-catenin complex can also initiate classic Wnt signaling transduction [32, 2]. These results indicate that typical Wnt and YAP cross talk through a variety of mechanisms. Compared with hGC33-null-NP and hGC33, hGC33-SFB-NP had stronger anti-proliferation and anti-migration ability in vitro and in vivo. Thus, hGC33 not only determines the specificity of HCC cells, but also increases the inhibitory effect of SFB on the proliferation and migration of HCC cells by blocking the key signals related to tumor growth, such as Wnt/β-catenin and Wnt/YAP signaling pathway.

## Conclusion

Antibody hGC33 to glypican-3, a membrane protein that is overexpressed on hepatocellular carcinoma cells, increased binding of sorafenib-loaded polyethylene glycol-b-PLGA polymer nanoparticles (hGC33-SFB-NP) to glypican-3 on the cancer cells. Administration of the antibody-modified nanoparticles synergistically inhibited Wnt-induced signal transduction and Ras/Raf/MAPK signaling pathway; hepatocellular carcinoma cells were arrested in G0/1 phase by down-regulation of cyclin D1 expression, thus attenuating cancer cell migration by inhibiting epithelial–mesenchymal transition. hGC33-SFB-NP inhibited the growth of liver cancer in vivo and improved the survival rate of tumor-bearing mice.

## Data Availability

Yes, all data have presented in the manuscript.

## Abbreviations

AKT/PKB: Protein kinase B
c-MET: HGFR: Hepatocyte growth factor receptor
EE%: Encapsulation efficiency %
EMT: Epithelial mesenchymal transition
FTIR: Fourier transform infrared spectroscopy
^1^H NMR: ^1^H-nuclear magnetic resonance spectroscopy
HCC: Hepatocellular carcinoma
LC%: Drug loading %
MAPK: Mitogen-activated protein kinases
PDI: Polydispersity index
PI3K: Phosphoinositide 3-kinase
pRAD51: Phospho-RAD51
SFB: Sorafenib
TEM: Transmission electron microscopy
YAP: Yes-associated protein-1
